# Are cultural dimensions relevant for explaining cross-national differences in antibiotic use in Europe?

**DOI:** 10.1186/1472-6963-8-123

**Published:** 2008-06-06

**Authors:** Reginald Deschepper, Larissa Grigoryan, Cecilia Stålsby Lundborg, Geert Hofstede, Joachim Cohen, Greta Van Der Kelen, Luc Deliens, Flora M Haaijer-Ruskamp

**Affiliations:** 1 Department of Medical Sociology and Health Sciences, Vrije Universiteit Brussel, Brussels, Belgium; 2 Department of Clinical Pharmacology, University Medical Center Groningen, University of Groningen, The Netherlands; 3 Division of International Health (IHCAR), Department of Public Health Sciences, Karolinska Institutet, Stockholm and Nordic School of Public Health and Apoteket AB, Göteborg, Sweden; 4 CentER for Economic Research, University of Tilburg, The Netherlands

## Abstract

**Background:**

Antibiotics are widely-used medicines for which a more prudent use has been advocated to minimize development of resistance. There are considerable cross-national differences that can only partially be explained by epidemiological difference and variations in health care structure. The aim of this study was to explore whether cross-national differences in use of antibiotics (prescribed and non-prescribed) are associated with differences between national cultures as described in Hofstede's model of cultural dimensions (Power Distance, Individualism, Masculinity, Uncertainty Avoidance and Long-Term Orientation).

**Methods:**

Country-level data of prescribed antibiotic use and self-medication with antibiotics were correlated to country-specific scores of cultural dimensions obtained from Hofstede. Data on use of antibiotics were provided by three European studies, based on different methods and/or countries: Self-medication with Antibiotics and Resistance in Europe (SAR), based on a survey in 2003 on reported use of antibiotics in 19 countries, the European Surveillance on Antimicrobial Consumption, based on distribution and reimbursement of antibiotics in ambulatory care (1997–2002), and the 2002 interview-based Eurobarometer study, asking whether respondents had taken antibiotics in the previous 12 months. These studies provided data on antibiotics use for 27 European countries in total, for which scores of cultural dimensions were also available. The SAR-study differentiated between prescribed antibiotics and self-medication with antibiotics.

**Results:**

Significant positive correlations were found for Power Distance Index with use of prescribed antibiotics in the three studies (rho between 0.59 and 0.62) and with self-medication (rho = 0.54) in the SAR study. Positive significant correlations were found for the Uncertainty Avoidance Index with the use of antibiotics as reported in two studies (rho between 0.57 and 0.59; for the SAR study the correlations were insignificant). Masculinity was not significantly correlated, except in one study after controlling for GDP (r = 0.81). For Individualism and Long-Term Orientation no significant correlations were found.

**Conclusion:**

Power Distance is a cultural aspect associated with antibiotic use, suggesting that the culture-specific way people deal with authority is an important factor in explaining cross-national differences in antibiotic use. There are indications that Uncertainty Avoidance also plays a role but further research is needed to better understand the complex effect of cultural dimensions.

## Background

Antibiotics are important and widely used medicines [1,2]. There is however a growing concern as the use of these medicines leads to resistance, avoidable costs and adverse reactions [1-3]. Hence it has been advocated to avoid unnecessary use [4].

Many studies have shown the relevance of characteristics of the doctor [5,6] or the patient [7,8], or the doctor-patient relationship [8-10] for the use of antibiotics. There are also considerable cross-national differences in public attitudes towards antibiotics use [7,11,12] as well as in actual use of prescribed antibiotics [2,13] and self-medication with antibiotics [14]. These cross-national differences in use of antibiotics can only partially be explained by epidemiological differences and variations in health care structure. Cultural factors also are known to play an important role in illness behaviour and consumption of antibiotics [11,12,15-17]. Most cross-cultural comparisons are about factors directly related to illness behaviour (e.g. labelling and presumed causes of illnesses). Few studies have explored the broader and general cultural characteristics of a country that at first sight are not directly related to illness behaviour [18]. One of the difficulties in many studies is that the concept of culture is often used as a general concept for all inexplicable cross-national differences. It is also hard to operationalise the concept of culture into quantifiable measures that can be used in comparative studies.

Several models and questionnaires have been developed to analyse cultural differences [19-21]. One of the most popular approaches is Hofstede's model of cultural dimensions [22]. These dimensions are aspects from which a culture can be compared with other cultures. They provide a relatively general framework for analysis that can be easily applied because it reduces the complexities of culture and its interactions into quantifiable dimensions [20,22,23]. Initially 4 cultural dimensions were found on which countries can be scored: Power Distance (PDI), Individualism (IDV), Masculinity (MAS) and Uncertainty Avoidance (UAI). Later the study has been replicated in other settings and other countries. Based on a Chinese Value Survey, a fifth dimension was found: Long-versus Short-Term Orientation (LTO) [20].

Power Distance refers to the degree of hierarchy in a country. It has been defined by Hofstede as the extent to which the less powerful members of organizations and institutions accept and expect that power is distributed unequally. A lower score indicates a preference for deliberation and mutual dependence between subordinates and their chief and authority based on secular/rational arguments. Individualism refers to the prevalence of the interests of an individual versus the group. This dimension is defined as the degree to which individuals are integrated into groups. A higher score indicates more emphasis on individualism. Masculinity, as opposed to femininity, refers to a culture in which the emotional roles of the two genders are clearly separated. A higher score indicates for instance that a culture is assertive and competitive. Uncertainty Avoidance deals with a society's tolerance for uncertainty and ambiguity. A higher score indicates that people feel uncomfortable in novel, unknown or surprising situations. Values associated with Long Term Orientation are thrift and perseverance; in contrast, a low score on this dimension implies values associated with Short Term Orientation: respect for tradition, fulfilling social obligations, protecting one's 'face' and expecting quick results.

While the influence of cultural dimensions on illness behaviour and use of medicines has been suggested in previous studies, [24,25] very few studies have actually linked the scores on cultural dimensions with diseases and illness behaviour [11,26].

Although antibiotics are used worldwide and their effectiveness is unquestionable, these medicines are the result of a particular view on illness, the so-called germ paradigm [27,28]. It incorporates some assumptions that were typical of a certain worldview in a particular (scientific) community and time, e.g. a reductionist approach assuming that a disease is caused by one (simple) cause: a germ. Within such a paradigm, antibiotics are ideal medicines. Alternatively, other approaches might put more emphasis on the inner resistance of the person or prefer 'to let nature run its course' [18]. If these worldviews and culture-specific assumptions, of which people are usually unaware and hence are seldom scrutinised, play a role, it becomes plausible that cultural dimensions also affect peoples' opinions, attitudes and use of antibiotics.

One of the authors (RD) postulated more that a decade ago what effect on antibiotic use could be expected of these cultural dimensions [29,30].

Power Distance has to do with preferences of how persons with a different status communicate with each other. Hence, one might expect that this will also play a role in patient-doctor communication. A preference for deliberation about the use and sense of antibiotics might be favoured in countries with low Power Distance, as contrasted with a 'doctor knows best'-attitude in countries scoring high for Power Distance.

Another hypothesis was that countries with high Uncertainty Avoidance would use more antibiotics because doctors try to avoid the ambiguity in cases of ailments such as 'a cold' or 'the flu', which are usually – but not always – trivial, self-limiting viral infections. Prescribing antibiotics is then a coping-strategy, much like a ritual, to reduce the uncertainty. In such countries patients too might have problems accepting a doctor saying no exact diagnosis can be given and therefore one should just 'wait and see'.

With regard to Individualism physicians face the problem that prescribing antibiotics for the individual patient increases the risk of resistance for the population as a whole. The same holds with regard to Long versus Short term Orientations: antibiotics are expected to give quick results but the prescribing of antibiotics now raises the risk of making them ineffective in the future.

Masculinity can be expected to correlate positively with the use of antibiotics, e.g. because these countries are strongly result-oriented and value a 'live to work' ethic. In such countries antibiotics might be favoured because they are often perceived and used as strong medicines that can get you back to work more quickly. Conversely, feminine cultures are more concerned with ecological problems and might therefore be expected to pay more attention to problems of antibiotic resistance.

Based on these hypotheses, it has been suggested that Power Distance and Uncertainty Avoidance were correlated with antibiotic consumption [16,17]. However, cultural aspects linked to the suboptimal use of antibiotics remained a point of particular interest calling for a better understanding [16].

The aim of this study is to test the aforesaid hypotheses and to further explore whether cultural dimensions are significantly correlated with cross-national differences in the prescribed use of antibiotics and self-medication with antibiotics.

## Methods

Data on the use of antibiotics were provided by three European studies, based on different methods and countries.

In the 'Self-medication with antibiotics and Resistance in Europe' (SAR) study, data were obtained from a survey in 2003 on the reported use of systemic antimicrobial drugs in the last year in 19 European countries. A multistage sampling design was used in each participating country. Within each country, a region with average prescribed antibiotic consumption was chosen, based on available national data on the use of medicines. However, in some countries these data were not available at the start of the SAR study, and a region representative of the country's population in terms of age and sex was chosen (Poland, Czech Republic, Lithuania, Croatia, Romania). In each region an urban area (population 75,000 – 350,000) and a rural area (population 5,000–10,000) were selected. Persons over 18 years of age were randomly selected from population registries in the selected regions. The questionnaire was developed specifically for this survey in English, translated into national languages, and back-translated for consistency. The SAR study could differentiate between prescribed antibiotics and self-medication with antibiotics. The questionnaire is available in English from the corresponding author. More information is provided elsewhere [14]. Respondents were classified as self-medicating if they reported that they had taken any antimicrobial drugs in the previous 12 months without a prescription from a physician, dentist, or nurse and as prescribed users if antimicrobial drugs had been prescribed. For all countries in the SAR-study with a response rate higher than 20%, prevalence of reported self-medication and prescribed use of antibiotics in the previous 12 months per 1000 population were estimated [14].

In the European Surveillance on Antimicrobial Consumption, data were based on either distribution or reimbursement of systemic antibiotics in ambulatory care for the period 1997–2002. Data were collected in 24 countries that provided internationally comparable data. Additional information about the characteristics of the data sources and data providers, obtained from the national ESAC representatives, was used to check validity of the data [2].

The Eurobarometer provided data based on face-to-face interviews of stratified at random samples of inhabitants, aged 15 years or older, in 15 countries. Samples consisted of at least 1000 persons per country (except for Luxemburg (N = 602) and Northern Ireland (N = 302)) and were compared to the total population. Respondents were asked whether or not they had taken antibiotics in the last 12 months [13].

Data on cultural dimensions were obtained from Hofstede [23]. For an initial set of 40 countries, Hofstede derived these indexes from carefully matched samples of employees in different national subsidiaries of the same multinational corporation IBM, between 1967 and 1973 [20]. Later additions used a simplified questionnaire, the Values Survey Module (VSM) 1994 [31]. It consisted of 14 content questions, scored on a five-point scale. The mean score for each country has been calculated, giving the index-value per country. The most recently published lists contain index values on the first four dimensions for 74 countries and regions [23]. Indexes refer to relative differences between countries. In the original study they varied between 0–100, but later, countries were added with higher scores. These findings have been replicated in a number of successive studies by different researchers using a variety of other matched samples of respondents [32]. For an overview see Hofstede, 2001 [20].

For the SAR study ethic or data committee approval was required and obtained for 11 countries [14]. For the other datasets we used data collected and published by other research teams.

Spearman's correlation coefficient rho was used to calculate correlations between country-aggregated use of antibiotics and country-specific scores of cultural dimensions. A p-value of 0.05 or smaller was considered significant. Since some cultural dimensions are known to covary with affluence [20,33], we examined the potential effects by calculating the partial correlations controlled for Gross Domestic Product per capita [34] and then compared the controlled correlation with the original correlation.

## Results

Two countries from the SAR study and two from the ESAC study could not be included because of lack of data on Hofstede's cultural dimensions (table 1). Hence correlations for 4 of the 5 cultural dimensions could be calculated for 27 European countries in total: 17 from the SAR study, 24 from the ESAC-study and 15 from the Eurobarometer study [2,13,14]. For Long-term versus short-term orientation, fewer correlations could be calculated because indexes on the latter dimension were available for only 18 of the 27 countries.

**Table 1:** Use of antibiotics and index scores on four cultural dimensions.

	SAR					CULTURAL DIMENSIONS^(e)^				
				
	No. Respondents^(a)^	Prescribed use of antibiotics^(b)^	Self-medication with antibiotics^(b)^	ESAC^(c)^	EURO BARO METER^(d)^	PDI	IDV	MAS	UAI	LTO
Sweden	704 (69%)	135	4			31	71	5	29	33
Netherlands	1634 (55%)	152	1	10	24	38	80	14	53	44
Austria	442 (28%)	159	9	12	34	11	55	79	70	31
Denmark	1881 (63%)	172	7	13	22	18	74	16	23	46
Poland	935 (32%)	199	33	21	-	68	60	64	93	32
UK	675 (23%)	221	12	14	40	35	89	66	35	25
Belgium (Fl.)	1734 (54%)	222	9	25	40	61	78	43	97	38
Czech Rep.	1169 (59%)	253	7	17	-	57	58	57	74	13
Lithuania	747 (25%)	275	210	-	-	42	60	19	65	-
Luxemburg	675 (50%)	288	9	27	41	40	60	50	70	-
Slovenia	1143 (38%)	293	17	17	-	71	27	19	88	-
Romania	430 (43%)	307	198	-	-	90	30	42	90	-
Ireland	793 (26%)	353	14	20	44	28	70	68	35	43
Malta	541 (54%)	422	56	-	-	56	59	47	96	-
Croatia	615 (31%)	439	31	22	-	73	33	40	80	-
Italy	213 (21%)	512	62	25	44	50	76	70	75	34
Slovakia	546 (55%)	569	42	22	-	104	52	110	51	38
Bulgaria	-	-	-	17	-	70	30	40	85	-
Estonia	-	-	-	12	-	40	60	30	60	-
Finland	-	-	-	18	36	33	63	26	59	41
France	-	-	-	32	45	68	71	43	86	39
Germany	-	-	-	13	26	35	67	66	65	31
Greece	-	-	-	30	40	60	35	57	112	-
Hungary	-	-	-	15	-	46	80	88	82	50
Norway	-	-	-	15	-	31	69	8	50	44
Portugal	-	-	-	27	44	63	27	31	104	30
Spain	-	-	-	19	45	57	51	42	86	19

(a) response rate between brackets

(b) use of antibiotics in the past 12 months, prevalence rate per 1000 respondents [14]

(c) total outpatient antibiotic use in DDD (defined daily dose) per 1000 inhabitants per day [2]

(d) percentage of persons who said they used antibiotics in the past 12 months [13]

(e) indexes of cultural dimensions [23].

Significant positive correlations were found for Power Distance Index with prescribed use of antibiotics in the three studies (with correlations varying from rho = 0.59 to 0.62; table 2).

**Table 2:** Correlations between use of antibiotics and cultural dimensions

	PDI	*p*-value	IDV	*p*-value	MAS	*p*-value	UIA	*p*-value	LTO	*p*-value
SAR Self-medication (N = 17)	**0.54**	*0.026*	-0.41	*0.104*	0.31	*0.228*	0.43	*0.086*	-0.12^g^	*0.722*
SAR Prescribed use (N = 17)	**0.59**	*0.012*	-0.45	*0.072*	0.42	*0.094*	0.32	*0.208*	-0.02^g^	*0.958*
ESAC (N = 24)	**0.59**	*0.003*	-0.29	*0.166*	0.22	*0.308*	**0.59**	*0.003*	-0.13^h^	*0.620*
Eurobarometer (N = 15)	**0.62**	*0.014*	-0.30	*0.280*	0.31	*0.259*	**0.57**	*0.025*	-0.39^i^	*0.190*

**Controlled for GDP/capita**										

SAR Self-medication (N = 17)	0.23 0.41	*0.615*	-0.03 0.10	*0.948*	0.61 0.58	*0.150*	0.19 0.26	*0.683*	0;00^g^	*0.998*
SAR Prescribed use (N = 17)		*0.362*		*0.831*		*0.172*		*0.572*	-0.01^g^	*0.977*
ESAC (N = 24)	0.72	*0.067*	0.09	*0.851*	0.38	*0.395*	0.55	*0.206*	-0.07^h^	*0.887*
Eurobarometer (N = 15)	0.50	*0.249*	0.10	*0.834*	**0.81**	*0.027*	0.50	*0.252*	-0.50^i^	*0.255*

g) based on 11 cases

h) based on 18 cases

i) based on 13 cases

Positive correlations were found for the Uncertainty Avoidance Index with the use of antibiotics as reported in the three studies (rho between 0.57 and 0.59). For the SAR study correlations are not significant, however.

For Individualism, Masculinity and Long-Term versus short-term Orientation the correlations were not significant.

Self-medication was only significantly correlated with Power Distance (rho = 0.54).

When controlling for the Gross Domestic Product (GDP) per capita, the correlations changed for Power Distance and Uncertainty Avoidance and became not significant. Correlations were also insignificant for Individualism and Long Term Orientation. For Masculinity the correlations tend to become stronger but they were significant for only one dataset (Eurobarometer: r = 0.81).

## Discussion

The findings of positive correlations for antibiotic use with Power distance and, in 2 of the 3 studies with Uncertainty Avoidance, corroborate our hypotheses with regard to these dimensions. When controlling the correlations between cultural dimensions and antibiotics for wealth, only one correlation (for Masculinity and the Eurobarometer results for antibiotic use) is significant.

Our findings may have implications with regard to the more rational use of antibiotics and might help us to improve our understanding of why there are such remarkable differences in antibiotic use. The effect of Power Distances is complex and might work on several levels. The preference for consultation and secular/rational arguments, which is typically of countries with low scores on Power Distance, may have implications for collaboration between doctors and other health care providers, such as pharmacists and nurses, as well as for doctor-patient communication. In countries with a high Power Distance, hierarchic differences are more manifested and doctors may therefore have a more pronounced (therapeutic) autonomy, whereas in other countries one can expect doctors to feel more part of a team. In the Netherlands (a country with a low Power Distance and one of the lowest use of antibiotics) Pharmacotherapy Counselling Groups, in which GPs and local community pharmacists regularly meet to exchange information about drug therapy, have been the spearhead of a successful policy for more rational use of antibiotics and other medicines [18,35,36].

Power Distance is also supposed to affect the doctor patient-relationship, as well as the relations between professionals. In countries with a high Power Distance it is likely that patients look up to their doctor, expecting great expertise and feeling little need for involvement in the decision-making. A doctor asking "what do you think yourself about taking an antibiotic?" would embarrass the 'modal' patient. Likewise, a physician acknowledging that he is not sure whether it is a viral or bacterial infection will not inspire much confidence in such a cultural context. Prescribing antibiotics has strong symbolic connotations and it can be seen as a sign of power and expertise [18]. Hence antibiotics have a communicative aspect as well as the pharmaceutical one. The clinician-patient encounter has been described as 'one of the most important battlegrounds' in the struggle with the microbe [12].

Countries with low Power Distance, in contrast, show a preference for shared decision-making in which the patient discloses his concerns and point of view, e.g. with regard to the pros and cons of antibiotics. It is true that patients often put pressure on the physician to prescribe antibiotics but doctors often overestimate patients demand [8]. Taking the time to talk about patients' concerns and to make patient expectations explicit has been promoted as a good strategy to attain a more rational use of antibiotics [10,37,38]. The finding that Power Distance is relevant with regard to use of antibiotics is in line with other studies [37] that indicate that doctor-patient relationship and dissatisfaction with the doctor is an important determinant of antibiotic misuse. Of four types of patients (involved, deferent, ignored and critical types), the involved patients, i.e. those who ensure they are part of the decision-making process and seeing themselves as equal to the doctor, have views on and usage of antibiotics most in line with the medical views [37]. Therefore, changing the patient to the more involved type is suggested as a strategy to limit the use of antibiotics. However this might be more difficult in countries with high Power Distance.

If we apply the avoidance of uncertainty to medical situations, patients and physicians are expected to have an aversion to diagnostic uncertainty and will prefer a clear labelling of diseases. However, many symptoms, such as coughing, sore throat, mild fever, etc. are difficult to label and remarkable cross-cultural variations have been found [24,39]. Based on a clinical examination of the patient, it is often impossible to determine whether the problem is caused by a (self-limiting) viral infection or a bacterial infection, in which cases antibiotics might be warranted [40]. Although many infections are self-limiting diseases and it is often good practice to 'wait and see', in cultures with a high avoidance of uncertainty, physicians might feel the inner urge 'to do something' and to fulfil the expected role of the expert who always has a solution. Hence they are more likely to start using antibiotics immediately, probably with a preference for the newest and strongest broad-spectrum antibiotics. When Uncertainty Avoidance is high, rather than returning home with the message that 'the doctor does not know' what disease they have or how to cure it, they accept the known risks of using antibiotics. In the latter case, patients feel confident that they have a disease with a clear cause that is under control. The above attitude of avoiding any risk is known as defensive medicine and leads to unnecessary high medical consumption [41].

Self-medication might be intuitively expected to be an expression of individualism. Talking F Rahme's saying into account "If I use an antibiotic too much, I am making it less useful for everyone" [42] we also should expect Individualism to be linked to higher use of antibiotics. Contrary to these expectations, we did not find a significant positive correlation for either self-medication or prescribed use of antibiotics. However, the inversion from negative to slightly positive correlations when controlling for GDP per capita suggests that the wealth of a country might have an effect on its use of antibiotics [20]. A hypothesis needing further exploration is that Individualism, in the sense that the interests of the individual prevail over the interests of the group, leads to higher use of antibiotics but that the effect on country-level is completely masked by the wealth of a country.

The results with regard to Long-term orientation are not conclusive, probably because of the limited number of countries for which correlation could be calculated.

We found few differences between the effects of cultural dimensions on prescribed use of antibiotics versus self-medication. This suggests that in countries with high incidence of self-medication with antibiotics, the prescription of antibiotics is also high [43] and that self-medication and prescribing are practices affected by the same cultural dimensions. This adds evidence to the hypothesis that, although the physician is formally the only person to decide whether or not antibiotics should be used, the final decision is the result of a complex interaction between patient and physician in a particular cultural context. There is no indication in our data that with regard to the use of antibiotics the behaviour of physicians is less dependent on the local cultural context than that of lay people (patients).

The finding that, after controlling for GDP/capita, the correlations become insignificant for Power Distance and Uncertainty Avoidance illustrates the complex relationship between a country's wealth and cultural dimensions. Also correlation values for Masculinity change. The original positive insignificant correlations become stronger (significantly for the Eurobarometer data) which is an indication of the complex relationship between wealth and cultural dimensions. Other studies have pointed out a possible effect of cultural dimensions on wealth and vice versa: Power Distances covaries negatively with a country's wealth. Uncertainty Avoidance influences consumer behaviour. Masculinity affects the way countries spent their money (e.g. masculine countries are inclined to prioritise economic growth over care for the environment and development aid). Long Term Orientation correlates with economic growth. [20,33]

Our study has some limitations. First, due to the selection of regions in the SAR study, it is not certain that the chosen regions really reflect the national culture of these countries. Also the response rate for the SAR data was low in some countries. We therefore repeated the analysis, limited to countries with response rates of >40%. We also plotted the results and checked for the effect of possible outliers. These verifications corroborated our results. We also used data from others studies, based on other data sources and sampling methods to minimise the biases and restrictions of each particular study. Secondly, since we tested correlations for 5 cultural dimensions the chance of finding statistically significant correlations by chance increases in one dataset. We used different data sets to test hypotheses that were formulated before the data were available. Nevertheless extra care is warranted in interpreting the results. Thirdly, culture is certainly a much richer phenomenon than the reduction into 5 dimensions presented and Hofstede has been criticized for trying 'to measure the immeasurable'. [44,45] However, equivalents of Hofstede's cultural dimensions have been found by several other authors and his cultural dimensions have been validated in multiple other studies [20,32]. Finally, as in most cross country comparisons the N is rather low, and the study may be underpowered to detect smaller differences between countries.

The VSM questionnaire has been developed to compare countries. It is not meant to compare individuals but to describe relative differences between social systems only. A common pitfall is to look at culture as a kind of collective personality. Such an interpretation neglects the fact that cultures are the result of interaction between different – at the same time conflicting and complementary – personalities [20]. Therefore, this study is done at the aggregated level of the country. The use of data aggregated at the country level requires extra caution to avoid the well-known ecological fallacy. Therefore, significant associations do not necessarily imply a causal relation.

We are also aware of the fact that cultural dimensions might be the resultant of important factors (as we showed for the impact of GDP). However, the strength of cultural dimensions is that they are the resultant of many interacting factors and that they can put phenomena together that initially seem unconnected. An advantage is also that they enable quantitative analysis of cultural aspects in relation to other relevant issues such as the use of antibiotics. This is not possible using thicker descriptions of cultures, i.e. anthropological descriptions based on qualitative data. Therefore, we believe cultural dimensions are a simple but useful tool to measure the extremely complex concept of culture. Up till now, this approach has seldom been used to improve our understanding of the remarkable and partially unexplained national differences in antibiotic us, but has been successfully employed in dozens of studies, e.g. on management and communication [46-48]. It provides a novel approach that may also be inspiring to explain other differences in the use of medical care.

## Conclusion

In summary, our study indicates that Power Distance is a cultural aspect associated with antibiotics use, suggesting that the culture-specific way people deal with authority is an important factor in explaining cross-national differences in antibiotic use. Uncertainty Avoidance can lead to high consumer demand and defensive medicine, which may result in unnecessary use of antibiotics. The effect of cultural dimensions is complex because they influence a multitude of social phenomena on the macro and micro level, such as the wealth of a country. Further cross-national comparative research, e.g. based on larger samples and including also non-Western countries, is needed to better understand the correlations between cultural dimensions and the use of antibiotics and the reasons for this, so that our hypothetical explanations can be corroborated or falsified.

## Competing interests

The authors declare that they have no competing interests.

## Authors' contributions

RD participated in the design of the study and data collection, carried out the analysis and drafted the manuscript. LG participated in the design of the study and data collection, the analysis and helped to draft the manuscript. CSL participated in the design of the study, the data collection and critically revised the manuscript. GH participated in the design of the study and critically revised the manuscript. JC participated in the design of the study, helped with the analysis and critically revised the manuscript. GVDK participated in the design of the study and the data collection, and critically revised the manuscript. LD participated in the design of the study and the data collection, and critically revised the manuscript. FMH–R participated in the design of the study and coordinated it, helped with the analysis and the drafting of the manuscript. All authors read and approved the final manuscript.

## Pre-publication history

The pre-publication history for this paper can be accessed here:


